# French Versions of 2 English Questionnaires on Problematic Digital Use Assessed by Adolescents and Their Parents: Cross-Cultural Linguistic Translation and Adaptation Study

**DOI:** 10.2196/55685

**Published:** 2025-07-09

**Authors:** Islam El Boudi, Mathilde Riant, Alexandre Bellier, Nicolas Vuillerme

**Affiliations:** 1 Faculty of Medicine, AGEIS (Autonomie, Gérontologie, E-santé, Imagerie et Société), Grenoble Alpes University, Jean Roget Building, 3rd Floor, Grenoble, 38000, France, 33 695685616; 2 Institut Universitaire de France, Paris, France; 3 Grenoble Clinical Investigation Center (Inserm CIC 1406), Grenoble Alpes University Hospital, Grenoble Alpes University, Grenoble, France

**Keywords:** internet addiction, screen time, questionnaire and surveys, translations, adolescents, mobile phone

## Abstract

**Background:**

The emergence of problematic digital use is increasingly alarming, affecting between 7% and 20% of the world’s adolescent population. However, there is no validated questionnaire in French to measure this. Only a few questionnaires, either self-reported by adolescents or hetero-reported by parents, have been translated and validated in English.

**Objective:**

This study aims to translate into French the Digital Addiction Scale for Children (DASC), which is self-reported by adolescents, and the Problematic Media Use Measure (PMUM), which is hetero-reported by parents of adolescents.

**Methods:**

We used the “forward and backward” method to establish the translation and achieve cross-cultural adaptation with 8 parents and 8 adolescents aged between 12 and 17 years. There were three stages: (1) initial translation and synthesis or reconciliation of the translations phase; (2) back translation and expert committee phase; and (3) pretesting phase, during which 8 parents completed the PMUM questionnaire and 8 adolescents completed the DASC questionnaire.

**Results:**

Despite slight variations in translation for both questionnaires, the translators quickly reached a consensus during the translation phase. The expert committee did not propose any other conceptual changes. In the final phase, the parents made no comments to improve the questions or the wording. Although some adolescents mentioned repetition between certain questions, they did not suggest any improvements to the DASC questionnaire in French.

**Conclusions:**

Although the translated versions of the DASC and PMUM questionnaires provide a foundation for detecting problematic digital use, they require further validation studies to confirm their reliability and applicability in the French adolescent population.

## Introduction

While smartphones make it easy to be entertained anywhere, computers, tablets, and video game consoles can also be a source of entertainment with negative impacts on health [1-3]. Meng et al [4] determined that 26% of the world’s population have problematic use of smartphones and 16% have problematic use of social networks. Internet gaming disorder, recognized in the *DSM-5* (*Diagnostic and Statistical Manual of Mental Disorders, Fifth Edition*) [5], affects 6% of the population, rising to 22% of French adolescents [4].

Today, there is no consensus for defining digital addiction, but some researchers agree to say that, as a behavioral addiction, it is characterized by intensive use of all types of digital content with difficulty in self-regulation [4]. This implies difficulty in being aware, planning, controlling, and evaluating its limits regarding this use, both in time and in content [4,6]. We will refer to this as problematic digital use (PDU). Among the consequences of the PDU, we find that major exposure to television or video games has deleterious effects on students’ attitudes toward school and is even linked to poorer academic performance [7,8]. In addition, this type of use is known to be linked to a lower sleep quantity and sleep quality [9,10], a more sedentary lifestyle [11], lower self-esteem, and more anxiety-depressive symptoms [10,12].

To estimate the prevalence of PDU, questionnaires are most often used in research, and some of them have already been validated in several countries and with several age profiles. To measure problematic smartphone use, we have identified 3 scales already validated in French. The first is the Internet Addiction Test (IAT)–smartphone [13] and is strongly inspired by the IAT developed by Young [14]. The second scale, Smartphone Addiction Scale short version [15], comprises 10 items and is based on the 6 symptoms present in the *DSM-5* to assess internet gaming disorder [5], such as loss of control or disruption of family. A final scale, Problematic Mobile Phone Use Questionnaire by Lopez-Fernandez et al [16], measures the same problem with 15 items divided into 3 dimensions: dangerous use, forbidden use, and dependent use of the smartphone. Three other questionnaires were translated and validated in French to assess problematic internet use. The IAT [17] has been validated on medical students, the Compulsive Internet Use Scale [18] is intended for adults, and the Generalized Problematic Internet Use Scale-2 [19] targets a general student population. Finally, the only questionnaire that measures problematic video game use in French is the Internet Gaming Disorder-20 [20], which has been validated with 20 items divided into 6 categories: salience, mood, tolerance, craving, conflict, and relapse.

To date, there exists no validated scale in French for quantifying digital addiction, and no measure has specifically addressed the adolescent population. However, adolescents are robust consumers of diverse digital content, perceiving it as a means to satisfy their autonomy needs and identity development, and to establish or maintain interpersonal connections [21,22]. While short-term benefits are highly valued by young people, the costs of intensive use are significant, leading to more risky behaviors, lower self-esteem, depressive symptoms, and suicidal ideation [23,24]. PDU can significantly alter the development of adolescents by causing neuronal dysfunctions in subcortical, frontal, and parietal areas involved in attention and behavioral control [21].

To measure PDU in adolescents, the English scale by Hawi et al [25], the Digital Addiction Scale for Children (DASC), is a promising tool. The scale is based on the 9 criteria recognized for diagnosing internet gaming disorder in the *DSM-5*. An existing alternative or complementary solution involves asking parents to assess their adolescents’ PDU. Domoff et al [26] proposed a questionnaire in 2 versions: the final versions of the Problematic Media Use Measure (PMUM; 27 items) and the PMUM Short Form (9 items). The PMUM has been validated in English and considers the 9 criteria of the *DSM-5* for internet gaming disorder. Although translated into English and Arabic, it has never been translated into French.

Whereas most questionnaires measure specific device use (eg, smartphone) [13,15,16], this study aims to better measure PDU in the French adolescent population by linguistically translating 2 questionnaires: the DASC, administered to adolescents, and the PMUM, administered to parents of adolescents. The translation process was carried out using the “forward and backward” method.

## Methods

### Ethical Considerations

This study was approved by the Grenoble Alpes University Hospital Center and has received ethics approval from the South-East I Ethics Committee for the Protection of Individuals (approval 2022-A01943-40). The collected data were anonymized throughout the analysis process, in compliance with the MR003 methodology approved by the National Commission on Informatics and Liberty. For minor participants, consent to participate was obtained from the teenagers and their parents. Adult participants gave their own consent. Furthermore, no compensation was provided to the participants.

### Cross-Cultural Adaptation Procedure With the “Forward and Backward” Method

#### Overview

We assessed the conceptual equivalence of the 2 questionnaires following the recommendations of Epstein et al [27] and used the most commonly adopted technique for cross-cultural research: the “forward and backward” method. Conceptual equivalence—evaluating whether items in the original language have a similar meaning in French—was emphasized by systematically analyzing item, semantic, idiomatic, operational, and experiential equivalence [28].

The forward and backward method is divided into 3 main phases of analysis, which we describe below.

#### Initial Translation and Synthesis or Reconciliation of the Translations Phase

We recruited 4 independent translators who are native French speakers and bilingual in English to translate the original French version. Two of the translators were familiar with the concept of PDU in their professional activities, while the other 2 did not work with this concept. From the 4 translations, the translators reconciled their differences to produce a single unified version.

#### Back Translation and Expert Committee Phase

The French version was back translated into English by a native English speaker to verify the accuracy of the translation and highlight any confusing wording. Then, 2 expert researchers familiar with the concept of PDU evaluated the equivalence of the questionnaire by comparing the back translated version with the original version. They proposed modifications to the items in French if necessary.

#### Pretesting Phase

We carried out a preliminary pilot study with adolescent participants and parents to assess the clarity, intelligibility, and acceptability of the French translations. The sampling was conducted using a convenience method through calls for participation in schools and libraries. A call for participation was shared during conference activities that we led about digital addiction. Participant criteria were specified to select specific settings such as age and region. Adolescents and parents responded to the call if they agreed to participate. Between December 2022 and June 2023, a total of 8 parents completed the PMUM questionnaire, and 8 adolescents aged between 12 and 17 years completed the DASC questionnaire. Accompanied by a student observer who cross-checked information to minimize interpretation biases, the principal researcher spoke with the participants, who gave their opinions on the questionnaires and assessed whether the questions were relevant to their age group. To categorize participants’ feedback, we conducted a qualitative thematic analysis by manually coding their comments into four categories: (1) linguistic incomprehension, (2) linguistic ambiguity, (3) suggestions for modification, and (4) redundancies.

The protocol consisted of an individual interview with each participant, who completed the questionnaires. Each interview lasted a maximum of 30 minutes.

### Instruments

The DASC questionnaire designed by Hawi et al [25] is composed of 25 statements distributed across 9 dimensions taken from the *DSM-5* video game addictive disorder criteria [5]: preoccupation in daily life, tolerance, withdrawal symptoms, mood changes, social and psychological consequences, relapses, physiological problems, deception regarding its use, and family disconnection. In the absence of the original online guideline that introduces the questionnaire to the participants, we took inspiration from the PMUM scale [26] by specifying that we want to capture adolescents’ experiences with their screens (ie, television, video games, tablets, smartphones, portable game consoles, laptops, or computers). The adolescent must reflect on his or her experiences over the past year. The response scale ranges from 1=never to 5=always, and the total score can vary from 25 to 125.

The PMUM questionnaire is composed of 27 statements and was designed by Domoff et al [26], who based the instrument on the diagnostic criteria for online gaming addiction disorder in the *DSM-5* [5]. The guideline consists of asking parents how their child uses screens (ie, television, video games, tablets, smartphones, portable game consoles, laptops, or computers). For each statement, they select the option that is true for their child during the last year. For each question, the response scale is from 1=never to 5=always; the total average score on this scale can vary from 1 to 5.

### Data Analysis

Data were analyzed using a qualitative approach with the forward and backward method, involving iterative interpretation of the interview responses. Descriptive statistics were conducted on the sample characteristics, such as age and gender. The qualitative analysis focused on identifying 4 key themes from the interviews.

## Results

### Characteristics of the Translators and the Participants

The age, gender, and specific cultural characteristics of the translators, the parent and adolescent participants are detailed in Table 1

**Table 1.** Age, gender, and cultural characteristics of the translators and the participants.

Step and participants	Age (years), mean (SD; range)	Gender	Cultural characteristics
Forward translation			
4 translators	25.5 (4.51; 20-31)	2 women and 2 men	2 native English speakers and 2 native French speakers 2 were familiar with the concept of problematic smartphone use (eHealth and psychology) 2 were not familiar with the concept (economics and education) 2 were from the south and 2 from the north of France
Backward translation			
1 translator	28	1 woman	Native English speaker No clinical or medical expertise
Linguistic verification			
2 experts	27.5 (4.95; 24-31)	1 woman and 1 man	2 native French speakers
Final verification			
8 parents	43.1 (2.59; 40-48)	4 women and 4 men	Good health and not experiencing any severe medical conditions 2 from rural areas and 6 from urban areas in France
8 adolescents	13.8 (1.83; 12-17)	6 girls and 2 boys	Good health or enduring any medical condition 3 from rural areas and 5 from urban areas in France 6 from middle school and 2 from high school

### Forward Translation

Four translators translated the titles, dimensions, guidelines, response categories, and statements of the 2 questionnaires. The 4 proposals were grouped to arrive at a consensual version. Although the titles, response categories, and guidelines did not pose a translation problem, there was less consensus on some dimension names. This mainly concerns the “withdrawal” dimension present in the 2 questionnaires, which has ultimately been translated as “sevrage.” The second, more problematic dimension to translate was that of the DASC scale: “displacement.” In the context of behavioral addiction, displacement corresponds to the implementation of intense or compulsive displacement behaviors when an individual is in a stressful situation [29]. We opted for the “déplacement” term that is adapted and used in French [30]. Similarly, for the DASC scale, although “device” is generally translated in French as “appareil,” we opted for “écrans” to better suit the context. For item 15, “enjoyable” offered multiple translation options, we translated it as “amusant” to adapt it to our context. For item 20, “I check my device…” had 4 different translations among the 4 translators as “to check” has a wide range of relevant translations. Therefore, we decided to translate it as “vérifier” even though it is not the most common word among all translation options, but as it is the most close translation from the original sentence without impacting the overall item meaning and conveying an intentional and repeating action. For item 25, “grades” was translated by “notes scolaires” as the raw translation “notes” would add too much ambiguity on the overall item.

For the PMUM questionnaire items, 23 of them were very similar between the 4 translations. Four items varied slightly for 1 or 2 translators. For instance, for item 10, the term “enjoys” offered multiple translation options; to achieve both semantic and idiomatic equivalence, we chose “apprécier,” resulting in: “Il n’y a rien que mon enfant n’apprécie autant que les écrans.” In 2 items, the term “amount of time” lacks a direct French equivalent. To preserve the operational and semantic meaning, we chose “la quantité de temps” for item 13 and “des périodes de plus en plus longues” for item 14. In item 22, the ambiguous term “sneaks” was resolved as “en cachette.”

As for the DASC questionnaire items, 22 reached consensus, and 3 items varied slightly for 1 or 2 translators. Although “device” is typically rendered as “appareil,” we selected “écrans” for better contextual equivalence. For item 15, to capture the intended semantic nuance of “enjoyable,” we chose “amusant.” In item 20, given the wide semantic range of “to check,” we opted for “vérifier” to best preserve the original meaning and indicate a repeated action. Finally, for item 25, “grades” was translated as “notes scolaires” to avoid ambiguity and ensure semantic clarity.

### Backward Translation and Linguistic Verification

After the questionnaires were translated from the consensus version into English by the native English translator, we observed that all 21 out of 27 items of the PMUM questionnaire and all 25 items of the DASC questionnaire were accurately retranslated into English. For the PMUM questionnaire, some formulations or terms varied but did not modify the meaning of the items. One item’s semantic meaning was strongly altered during the back translation: “My child attempts to use screen media for increasing amounts of time.” was retranslated as “My child keeps using screens for longer periods.” At the semantic level, this back translation does not reflect the intention of using the screen more and more—as covered by the initial item. Consequently, we modified the French item translation to better capture the original meaning of the item.

After these modifications, 2 researchers reread the latest French translation during a final professional translation step. No suggestions or conceptual modifications were proposed for the 2 questionnaires.

### Final Verification

The final versions of the PMUM and DASC questionnaires were delivered to adolescents and parents in the pilot study. The 8 parents had no comments for improvement for any of the items or the statements, including linguistic incomprehension, linguistic ambiguity, suggestions for modification, or redundancies.

Although some adolescents noted repetitive phrasing (redundancies) across items addressing withdrawal symptoms—for example, items 12 and 21 use “frustrated” while items 3 and 8 use “upset,” reflecting potential linguistic ambiguity—the expert team determined that these repetitions were essential for capturing subtle distinctions in behavior and experience, ensuring a comprehensive assessment of the construct, even if the phrasing occasionally feels redundant to adolescents, because “frustrated” and “upset” have close semantic meaning in French. No linguistic incomprehension was reported at this stage, and this feedback did not lead to suggestions for modification of the 25 items of the DASC questionnaire. The final translated versions are listed in Multimedia Appendix 1.

## Discussion

### Principal Findings

We effectively conducted a cross-cultural adaptation of 2 original questionnaires into French to measure PDU among adolescents. These questionnaires are intended to be filled out by adolescents themselves (DASC) or by their parents (PMUM). Although the DASC and PMUM questionnaires were originally validated in children aged 9‐12 and 4‐11 years, we applied these instruments to adolescents aged 12‐17 years. One of the advantages of considering these 2 questionnaires is that, given that PDU in adolescents may manifest as extreme resistance to limits imposed by parents, leading to significant intrafamilial conflicts, questioning parents in addition to adolescents provides valuable information to better understand the extent of PDU. To make these questionnaires accessible in French, we translated them from English using the forward and backward method by following the 3 stages of translation of the method recommended by Epstein et al [27] and that has already been used for prior related French translations [31]. The conceptual equivalence of the French questionnaires was close to that of the English questionnaires. Regarding our results, neither parents nor adolescents encountered major difficulties in understanding the items, and experts recognized that the final translations were equivalent to the original questionnaires. Limitations include the small sample size, although the minimum number of participants for linguistic validation is not clearly defined [28], and the lack of validation. Further studies are required to validate these translations in larger populations and confirm their psychometric properties, particularly since adolescents reported that some items of the DASC questionnaire were redundant. Although other cross-cultural adaptations studies incorporated additional steps, such as confirmatory and exploratory factor analyses, to examine the instrument’s internal structure and reliability in a simulated clinical setting [32-34], our study did not include these analyses. Yet, as the DASC questionnaire aims to assess PDU—a construct that is not directly observable and is composed of multiple dimensions such as tolerance, withdrawal symptoms, and overuse or physical and psychological consequences—future research should further investigate its structure validity. Additionally, researchers will need to assess the convergent validity of the questionnaires by showing that the scores obtained from the 2 translated questionnaires are positively correlated with other similar or closely related measures. These questionnaires can also be supplemented by objective and passive measurements derived directly from digital tools [35]. The last step will consist of evaluating the reliability of the questionnaires about their consistency and stability with longitudinal test-retest analyses.

These questionnaires, validated in French, will be useful in research to make intercultural comparisons in PDU demographics. Additionally, they can be used for professional purposes as part of medical or psychological care to detect PDU among adolescents.

### Conclusions

This study enabled the effective cross-cultural adaptation of 2 questionnaires on the PDU of adolescents into French.

This study revealed that parents and adolescents easily understood the items, and experts confirmed the final translations’ equivalence with the originals. Once validation of the questionnaires in French is completed, these 2 questionnaires can be used in French-speaking regions to screen or measure PDU among adolescent populations. Limitations of this research include the small sample size and the lack of psychometric validation, which are necessary to establish the reliability and validity of the translated questionnaires.

This study provides a questionnaire enabling adolescents to assess their own digital addiction (DASC), and another on parents’ perceptions of their children’s use (PMUM), which will enable the research community to better detect or assess the prevalence of the PDU in the French adolescent population.

## Supplementary material

10.2196/55685 Multimedia Appendix 1 DASC and PMUM questionnaires. DASC: Digital Addiction Scale for Children; PMUM: Problematic Media Use Measure.

10.2196/55685 Multimedia Appendix 2 ChatGPT conversation history.

## Abbreviations

DASC: Digital Addiction Scale for Children
*DSM-5*: *Diagnostic and Statistical Manual of Mental Disorders, Fifth Edition*
IAT: Internet Addiction Test
PDU: problematic digital use
PMUM: Problematic Media Use Measure

## References

[1] Lissak G (2018). Adverse physiological and psychological effects of screen time on children and adolescents: literature review and case study. Environ Res.

[2] Wacks Y, Weinstein AM (2021). Excessive smartphone use is associated with health problems in adolescents and young adults. Front Psychiatry.

[3] Zhang Y, Tian S, Zou D, Zhang H, Pan CW (2022). Screen time and health issues in Chinese school-aged children and adolescents: a systematic review and meta-analysis. BMC Public Health.

[4] Meng SQ, Cheng JL, Li YY (2022). Global prevalence of digital addiction in general population: a systematic review and meta-analysis. Clin Psychol Rev.

[5] (2013). Diagnostic and Statistical Manual of Mental Disorders (DSM-5-TR). American Psychiatric Association.

[6] LaRose R, Lin CA, Eastin MS (2003). Unregulated internet usage: addiction, habit, or deficient self-regulation?. Media Psychol.

[7] Adelantado-Renau M, Moliner-Urdiales D, Cavero-Redondo I, Beltran-Valls MR, Martínez-Vizcaíno V, Álvarez-Bueno C (2019). Association between screen media use and academic performance among children and adolescents: a systematic review and meta-analysis. JAMA Pediatr.

[8] Johnson JG, Cohen P, Kasen S, Brook JS (2007). Extensive television viewing and the development of attention and learning difficulties during adolescence. Arch Pediatr Adolesc Med.

[9] Chen YL, Gau SSF (2016). Sleep problems and internet addiction among children and adolescents: a longitudinal study. J Sleep Res.

[10] Ratan ZA, Parrish AM, Zaman SB, Alotaibi MS, Hosseinzadeh H (2021). Smartphone addiction and associated health outcomes in adult populations: a systematic review. Int J Environ Res Public Health.

[11] Zagalaz-Sánchez ML, Cachón-Zagalaz J, Sánchez-Zafra M, Lara-Sánchez A (2019). Mini review of the use of the mobile phone and its repercussion in the deficit of physical activity. Front Psychol.

[12] Prizant-Passal S, Shechner T, Aderka IM (2016). Social anxiety and internet use – a meta-analysis: what do we know? What are we missing?. Comput Human Behav.

[13] Barrault S, Durousseau F, Ballon N, Réveillère C, Brunault P (2019). Smartphone addiction: French validation of the Internet Addiction Test-Smartphone Version (IAT-smartphone) and associated psychopathological features. Encephale.

[14] Young KS (1998). Internet addiction: the emergence of a new clinical disorder. Cyberpsychol Behav.

[15] Lopez-Fernandez O (2017). Short version of the Smartphone Addiction Scale adapted to Spanish and French: towards a cross-cultural research in problematic mobile phone use. Addict Behav.

[16] Lopez-Fernandez O, Kuss DJ, Pontes HM (2018). Measurement invariance of the Short Version of the Problematic Mobile Phone Use Questionnaire (PMPUQ-SV) across eight languages. Int J Environ Res Public Health.

[17] Khazaal Y, Billieux J, Thorens G (2008). French validation of the Internet Addiction Test. Cyberpsychol Behav.

[18] Khazaal Y, Chatton A, Horn A (2012). French validation of the Compulsive Internet Use Scale (CIUS). Psychiatr Q.

[19] Laconi S, Kaliszewska-Czeremska K, Tricard N, Chabrol H, Kuss DJ (2018). The Generalized Problematic Internet Use Scale-2 in a French sample: psychometric evaluation of the theoretical model. L’Encéphale.

[20] Plessis C, Altintas E, Romo L, Guerrien A (2021). French validation of a scale evaluating internet gaming disorder: the Internet Gaming Disorder-20. Can J Psychiatry.

[21] Marciano L, Ostroumova M, Schulz PJ, Camerini AL (2021). Digital media use and adolescents’ mental health during the COVID-19 pandemic: a systematic review and meta-analysis. Front Public Health.

[22] Uhls YT, Ellison NB, Subrahmanyam K (2017). Benefits and costs of social media in adolescence. Pediatrics.

[23] Vannucci A, Simpson EG, Gagnon S, Ohannessian CM (2020). Social media use and risky behaviors in adolescents: a meta-analysis. J Adolesc.

[24] Vidal C, Lhaksampa T, Miller L, Platt R (2020). Social media use and depression in adolescents: a scoping review. Int Rev Psychiatry.

[25] Hawi NS, Samaha M, Griffiths MD (2019). The Digital Addiction Scale for Children: development and validation. Cyberpsychol Behav Soc Netw.

[26] Domoff SE, Harrison K, Gearhardt AN, Gentile DA, Lumeng JC, Miller AL (2019). Development and validation of the Problematic Media Use Measure: a parent report measure of screen media “addiction” in children. Psychol Pop Media Cult.

[27] Epstein J, Santo RM, Guillemin F (2015). A review of guidelines for cross-cultural adaptation of questionnaires could not bring out a consensus. J Clin Epidemiol.

[28] Harachi TW, Choi Y, Abbott RD, Catalano RF, Bliesner SL (2006). Examining equivalence of concepts and measures in diverse samples. Prev Sci.

[29] Anselme P (2008). Abnormal patterns of displacement activities: a review and reinterpretation. Behav Processes.

[30] Chabrol H, Callahan S (2018). Mécanismes de Défense et Coping.

[31] El Boudi I, Riant M, Bellier A, Vuillerme N (2025). French versions of 4 English questionnaires on problematic smartphone use: cross-cultural linguistic translation and adaptation study. Interact J Med Res.

[32] Bellier A, Chaffanjon P, Krupat E, Francois P, Labarère J (2019). Cross-cultural adaptation of the 4-habits coding scheme into French to assess physician communication skills. PLoS ONE.

[33] Zhao H, Rafik-Galea S, Fitriana M, Song TJ (2022). Translation and psychometric evaluation of Smartphone Addiction Scale-Short Version (SAS-SV) among Chinese college students. PLoS ONE.

[34] Adawi M, Bragazzi NL, Argumosa-Villar L (2018). Translation and validation of the nomophobia questionnaire in the Italian language: exploratory factor analysis. JMIR mHealth uHealth.

[35] Ryding FC, Kuss DJ (2020). Passive objective measures in the assessment of problematic smartphone use: a systematic review. Addict Behav Rep.

[36] (2023). ChatGPT (GPT-4). OpenAI.

