# Supplementary material for: French Versions of 2 English Questionnaires on Problematic Digital Use Assessed by Adolescents and Their Parents: Cross-Cultural Linguistic Translation and Adaptation Study
Source: Interact J Med Res. 2025 Jul 9;14:e55685. doi: 10.2196/55685 (PMC12266299; doi:10.2196/55685)
Supplement: Multimedia Appendix 1 — DASC and PMUM questionnaires. DASC: Digital Addiction Scale for Children; PMUM: Problematic Media Use Measure. [file ijmr-v14-e55685-s001.docx]

**Table S1.** Digital Addiction Scale for Children (DASC) translation.

|  |  | 1^a^ | 2^b^ | 3^c^ | 4^d^ | 5^e^ |
| --- | --- | --- | --- | --- | --- | --- |
| [1] | ET: When I am not at school, I spend a lot of time using my device.  FT: Quand je ne suis pas à l’école, je passe beaucoup de temps à utiliser mes écrans. |  |  |  |  |  |
| [2] | ET: I feel the need to spend more time using my device.  FT: Je ressens le besoin de passer plus de temps à utiliser mes écrans. |  |  |  |  |  |
| [3] | ET: I feel upset when I am not able to use my device.  FT: Je me sens contrarié.e quand je ne peux pas utiliser mes écrans.. |  |  |  |  |  |
| [4] | ET: I lie to my parents about the amount of time I spend using my device.  FT: Je mens à mes parents à propos du temps que je passe à utiliser mes écrans. |  |  |  |  |  |
| [5] | ET: Using my device helps me to forget my problems.  FT: Utiliser mes écrans m’aide à oublier mes problèmes. |  |  |  |  |  |
| [6] | ET: I do not spend time with my family members because I prefer using my device.  FT: Je ne passe pas de temps avec les membres de ma famille parce que je préfère utiliser mes écrans. |  |  |  |  |  |
| [7] | ET: I have spent more and more time on my device.  FT: Je passe de plus en plus de temps sur mes écrans. |  |  |  |  |  |
| [8] | ET: I feel upset when I am asked to stop using my device.  FT: Je me sens contrarié.e lorsque l’on me demande d’arrêter d’utiliser mes écrans. |  |  |  |  |  |
| [9] | ET: My parents try to stop or limit me using my device, but they fail.  FT: Mes parents essaient de m’arrêter ou me limiter dans l’utilisation de mes écrans, mais ils échouent. |  |  |  |  |  |
| [10] | ET: I am sleeping less because I am using my device.  FT: Je dors moins parce que j’utilise mes écrans. |  |  |  |  |  |
| [11] | ET: When I do not have my device, I think about what I do on it (video games, social media, and texting, etc.).  FT: Quand je n’ai pas mes écrans, je pense à ce que je fais dessus (jeux vidéo, réseaux sociaux, messagerie, etc.). |  |  |  |  |  |
| [12] | ET: I feel frustrated when I cannot use my device.  FT: Je me sens frustré.e quand je ne peux pas utiliser mes écrans. |  |  |  |  |  |
| [13] | ET: I have problems with my parents about the amount of time I spend using my device.  FT: J’ai des problèmes avec mes parents à propos du temps que je passe à utiliser mes écrans. |  |  |  |  |  |
| [14] | ET: Using my device is the most important thing in my life.  FT: Utiliser mes écrans est la chose la plus importante dans ma vie. |  |  |  |  |  |
| [15] | ET: Using my device is more enjoyable than doing other things.  FT: Utiliser mes écrans est plus amusant que de faire d’autres choses. |  |  |  |  |  |
| [16] | ET: I lie to my parents about what I do on my device.  FT: Je mens à mes parents à propos de ce que je fais sur mes écrans. |  |  |  |  |  |
| [17] | ET: I am not able to control using my device.  FT: Je ne suis pas capable de contrôler mon utilisation des écrans. |  |  |  |  |  |
| [18] | ET: I have lost interest in hobbies or other activities because I prefer using my device.  FT: J’ai perdu de l’intérêt dans les loisirs ou autres activités parce que je préfère utiliser mes écrans. |  |  |  |  |  |
| [19] | ET: When I stop using my device, it is not long before I start using it again.  FT: Quand j’arrête d’utiliser mes écrans, il s’écoule peu de temps avant que je les utilise de nouveau. |  |  |  |  |  |
| [20] | ET: I check my device when I am doing homework or other important things.  FT: Je vérifie mes écrans quand je fais mes devoirs ou d’autres choses importantes. |  |  |  |  |  |
| [21] | ET: I feel frustrated when I am asked to stop using my device.  FT: Je me sens frustré.e quand on me demande d’arrêter d’utiliser mes écrans. |  |  |  |  |  |
| [22] | ET: I argue with my parents when they ask me to stop using my device.  FT: Je me dispute avec mes parents quand ils me demandent d’arrêter d’utiliser mes écrans. |  |  |  |  |  |
| [23] | ET: I spend too much money on things for my device.  FT: Je dépense beaucoup trop d’argent pour des choses pour mes écrans. |  |  |  |  |  |
| [24] | ET: Using my device makes me feel better when I feel bad.  FT: Utiliser mes écrans me fait sentir mieux quand je me sens mal. |  |  |  |  |  |
| [25] | ET: I continue using my device despite the fact that my grades at school are getting lower and lower.  FT: Je continue d’utiliser mes écrans malgré le fait que mes notes scolaires deviennent de plus en plus basses. |  |  |  |  |  |

^a^English translation (ET): never -> French translation (FT): jamais.

^b^English translation (ET): rarely -> French translation (FT): rarement.

^c^English translation (ET): sometimes -> French translation (FT): parfois.

^d^English translation (ET): often -> French translation (FT): souvent.

^e^English translation (ET): always -> French translation (FT): toujours.

**Table S2.** Problematic Media Use Measure (PMUM) translation.

|  |  | 1^a^ | 2 | 3 | 4 | 5^b^ |
| --- | --- | --- | --- | --- | --- | --- |
| [1] | ET: It is hard for my child to stop using screen media.  FT: C’est dur pour mon enfant d’arrêter d’utiliser les écrans. |  |  |  |  |  |
| [2] | ET: It is increasingly difficult to pull my child away from screen media.  FT: C’est de plus en plus difficile de tenir mon enfant éloigné des écrans. |  |  |  |  |  |
| [3] | ET: It is really difficult to get my child to stop using screen media.  FT: C’est très difficile de faire arrêter mon enfant d’utiliser les écrans. |  |  |  |  |  |
| [4] | ET: Screen media is the only thing that seems to motivate my child.  FT: Les écrans sont la seule chose qui semblent motiver mon enfant. |  |  |  |  |  |
| [5] | ET: My child is always thinking about using screen media.  FT: Mon enfant est toujours en train de penser à utiliser les écrans. |  |  |  |  |  |
| [6] | ET: Screen media is all that my child seems to think about.  FT: Les écrans sont la seule chose à laquelle mon enfant sembler penser. |  |  |  |  |  |
| [7] | ET: My child becomes frustrated when he/she cannot use screen media.  FT: Mon enfant devient frustré lorsqu’il/elle ne peut pas utiliser les écrans. |  |  |  |  |  |
| [8] | ET: My child’s screen media use interferes with family activities.  FT: L’utilisation des écrans de mon enfant interfère avec les activités familiales. |  |  |  |  |  |
| [9] | ET: My child gets upset when he/she cannot use screen media.  FT: Mon enfant devient contrarié lorsqu’il/elle ne peut pas utiliser les écrans. |  |  |  |  |  |
| [10] | ET: There is nothing my child enjoys as much as screen media.  FT: Il n’y a rien que mon enfant n’apprécie autant que les écrans. |  |  |  |  |  |
| [11] | ET: My child becomes angry when he/she cannot use screen media.  FT: Mon enfant devient en colère lorsqu’il/elle ne peut pas utiliser les écrans. |  |  |  |  |  |
| [12] | ET: My child’s screen media use causes problems for the family.  FT: L’utilisation des écrans de mon enfant cause des problèmes pour la famille. |  |  |  |  |  |
| [13] | ET: The amount of time my child wants to use screen media keeps increasing.  FT: La quantité de temps que veut passer mon enfant sur les écrans continue sans cesse d’augmenter. |  |  |  |  |  |
| [14] | ET: My child attempts to use screen media for increasing amounts of time.  FT: Mon enfant utilise les écrans pendant des périodes de plus en plus longues. |  |  |  |  |  |
| [15] | ET: Problems occur for our family when my child cannot use screen media.  FT: Des problèmes surviennent dans notre famille lorsque mon enfant ne peut pas utiliser les écrans. |  |  |  |  |  |
| [16] | ET: My child would find life boring without screen media.  FT: Mon enfant trouverait la vie ennuyante sans les écrans. |  |  |  |  |  |
| [17] | ET: Life would be easier if my child was not so attached to screen media.  FT: La vie serait plus facile si mon enfant n’était pas aussi attaché aux écrans. |  |  |  |  |  |
| [18] | ET: The first thing my child asks to do when he/she comes home from school is to use screen media.  FT: La première chose que mon enfant demande à faire lorsqu’il/elle revient de l’école est d’utiliser les écrans. |  |  |  |  |  |
| [19] | ET: My child’s screen media use negatively affects his/her friendships.  FT: L’utilisation des écrans de mon enfant affecte négativement ses relations amicales. |  |  |  |  |  |
| [20] | ET: My child uses screen media for increasing amounts of time.  FT: Mon enfant passe de plus en plus de temps sur les écrans. |  |  |  |  |  |
| [21] | ET: My child loses sleep due to screen media use.  FT: Mon enfant perd le sommeil à cause de son utilisation des écrans. |  |  |  |  |  |
| [22] | ET: My child sneaks using screen media.  FT: Mon enfant utilise les écrans en cachette. |  |  |  |  |  |
| [23] | ET: My child lies about doing chores or school work in order to use screen media.  FT: Mon enfant ment à propos des tâches de la maison ou du travail scolaire afin d’utiliser les écrans. |  |  |  |  |  |
| [24] | ET: When my child has had a bad day, screen media seems to be the only thing that helps him/her feel better.  FT: Quand mon enfant a eu une mauvaise journée, les écrans semblent être la seule chose qui l’aide à se sentir mieux. |  |  |  |  |  |
| [25] | ET: My child feels better when he/she uses screen media.  FT: Mon enfant se sent mieux lorsqu’il/elle utilise les écrans. |  |  |  |  |  |
| [26] | ET: My child uses screen media to feel better.  FT: Mon enfant utilise les écrans pour se sentir mieux. |  |  |  |  |  |
| [27] | ET: My child lies in order to use screen media.  FT: Mon enfant ment afin d’utiliser les écrans. |  |  |  |  |  |

^a^English translation (ET): never -> French translation (FT): jamais.

^b^English translation (ET): always -> French translation (FT): toujours.
